# Knowledge and behaviors of prevention of COVID-19 and the related factors in the rural population referred to the health centers: a cross-sectional study

**DOI:** 10.1186/s12912-023-01469-5

**Published:** 2023-12-13

**Authors:** Fatemeh Kordi, Nasrin Mokhtari Lakeh, Moluk Pouralizadeh, Saman Maroufizadeh

**Affiliations:** 1 Department of Nursing, School of Nursing and Midwifery, Guilan University of Medical Sciences, Rasht, Iran; 2 Department of Nursing, School of Nursing and Midwifery, Guilan University of Medical Sciences, Rasht, Iran; 3 Department of Biostatistics and Epidemiology, School of Health, Guilan University of Medical Sciences, Rasht, Iran

**Keywords:** Coronavirus disease 2019, Behavior, Prevention, Knowledge

## Abstract

**Background and objective:**

Observance of preventive behaviors is one of the main ways to break the coronavirus disease 2019 (COVID-19) chain of transmission. Therefore, the present study was conducted to determine the knowledge and behaviors of prevention of COVID-19 and the related factors in the rural population of Rasht city.

**Methods:**

In this cross-sectional study, 344 people of the population referred to health centers in Rasht city were included through multi-stage cluster random sampling. The data were collected using a three-part researcher-made questionnaire including individual-social factors, knowledge about the prevention of COVID-19, and the preventive behaviors against COVID-19. Data analysis was performed using the Kolmogorov-Smirnov and Shapiro-Wilk tests, Spearman’s correlation coefficient, and multiple logistic regression analysis, by SPSS software version 16 at a significance level < 0.05.

**Results:**

The mean total score of knowledge about COVID-19 was at a moderate level, and the preventive behaviors of COVID-19 were at a good level. There was no significant relationship between the scores of awareness and preventive behaviors of COVID-19 (r_s_=0.001, P = 0.998). Awareness of COVID-19 was higher in university-educated individuals and women. Also, women, individuals who had access to the Internet, those trained by health centers, and those who were visited by health workers at home had more preventive behaviors.

**Conclusion:**

Despite the lack of connection between knowledge and preventive behaviors, the villagers living in the suburbs of Rasht had a moderate level of knowledge and a good level of preventive behaviors of COVID-19. Appropriate educational interventions should be carried out to increase the awareness and performance of the rural residents.

**Supplementary Information:**

The online version contains supplementary material available at 10.1186/s12912-023-01469-5.

## Background

Coronavirus disease 2019 (COVID-19) is an infectious disease caused by the new beta coronavirus [1, 2]. This disease is highly contagious [2], and its primary clinical symptoms include fever, dry cough, fatigue, shortness of breath, and muscle pains [2–4]. The most important way of transmission of this disease is from human to human through respiratory droplets, touch, or contact with an infected individual or surface [5–7]. The first case of COVID-19 was reported in December 2019 in Wuhan, China [8]. Considering the contagious nature and rapid spread of COVID-19 in different regions of the world, the World Health Organization (WHO) declared it a global pandemic on March 11, 2020 [9]. After China, Iran was one of the first countries in which the COVID-19 disease spread [10]. According to the WHO report on January 11, 2022, the total number of individuals infected with COVID-19 was more than 304 million cases, and about 6 million deaths occurred in 222 countries, of which, 131,940 cases were reported in Iran [11, 12].

With the spread of COVID-19 in Iran, extensive consequences were created for people’s health and livelihood, and daily life activities were disrupted [13]. The end of this disease is uncertain [13], and although vaccines have been produced to prevent the disease and universal vaccination has been carried out, there are the reports of cases of death and epidemic waves all over the world, and in fact, there is still no effective medicine for its definite treatment or prevention [14, 15]. This disease can also be transmitted through asymptomatic individuals [16]. Therefore, the most important and main way to control and prevent the spread of this disease is to eliminate the virus chain of transmission [10] and perform preventive behaviors [17].

Preventive health behaviors refer to any activity performed by a person who considers him/herself healthy to prevent disease [18]. These measures include home quarantine, isolation, limiting travel and commuting, physical and social distancing, staying away from gatherings, opening doors and windows in closed places, disinfecting surfaces, washing hands frequently, using face masks, observing the customs of sneezing and coughing, avoiding touching the eyes, mouth, and nose, and avoiding shaking hands and hugging [19–21].

Despite preventive measures, most people are severely affected by COVID-19 disease [13]. During this period, the rural population experienced the most damage to their livelihood due to disruption in the marketing and sale of agricultural products and extensive loss of jobs and incomes of informal workers. In other words, COVID-19 has had major effects on the villagers’ income and the village economy [12]. One of the reasons for the vulnerability of rural communities is that individuals in rural areas are much less ready to deal with the direct and indirect impacts of the COVID-19 crisis because villagers usually face a lower socioeconomic status, limited educational opportunities, and a lack of healthcare access [13].

It should be noted that preventive behaviors and public obedience to these behaviors are influenced by numerous physical, psychological, political, social, and cultural factors [10]. Also, many studies have shown that demographic characteristics are related to preventive behaviors during an epidemic. For example, the results of Khazaee-Pool et al.’s study conducted in Mazandaran province showed that age, gender, occupation, education level, place of residence, and history of contracting COVID-19 in the individual and at least one family member are related to performing preventive behaviors against COVID-19 [22].

As one of the northern tourism regions of the country, Guilan province has been facing severe disease crises during the recent epidemics, so that it has received special attention from the country’s health system as one of the most common areas of getting infected with COVID-19 disease with a high number of hospitalizations and deaths of patients [23]. Therefore, the present study was carried out to determine the rate of preventive behaviors against COVID-19 and its related factors in the rural population covered by health centers in the city of Rasht.

## Materials and methods

### Research design and setting

This cross-sectional study was performed on people who referring to health centers in Rasht, Iran.

### Participants and sampling

The participants were the population who were referred to the of affiliated to Rasht city. A multi-stage cluster random method was used for sampling and the participants were entered to the study from November to December 2021. Inclusion criteria included interest in participating in the study, not being infected with COVID-19 disease at the time of sampling, having over 18 years of age, and having literacy. The incomplete questionnaires were excluded. As a rule of thumb, in regression analyses, for each independent variable (predictor), at least 20 subjects (10 or 15 subjects in some sources) should be selected [24]. Considering the 17 independent variables in this study, the sample size was calculated as 344 participants.

### Data collection

The Rasht city have six rural health centers that provide health services to the people of 28 villages. For sampling, each health center was considered as clusters, and then according to the determined sample size and the geographical locations of the villages, 12 villages were randomly selected from these clusters and then a convenience sampling was done. Before sampling, the researcher explained about the research aims, the confidentiality of the information, and the voluntary participation in the study.

The data collection tool was a three-part researcher-made questionnaire that included, individual-social factors, knowledge of prevention of COVID-19 disease, and assessing preventive behaviors against COVID-19. This questionnaire was designed from the official national protocols of the Ministry of Health of Iran and the guidelines of the American Centers for Disease Control and Prevention (CDC).

The individual-social factors questionnaire had 16 questions included age, gender, marital status, number of family members, education level, occupation, family income status, underlying diseases, history of contracting COVID-19, loss of family members due to COVID-19, getting COVID-19 vaccine, access to health medical services in the village, accessibility to and using the Internet, receiving education about COVID-19 disease and ways to prevent it by the health center, the method of obtaining information related to COVID-19, and visiting health officers at home.

The second part of the study instrument included 11 questions about knowledge of prevention of COVID-19 based on the national protocols. A correct answer was given a score of 1, and a wrong answer was given a score of 0. The minimum score of this questionnaire was 0, and the maximum was 11. A higher score indicated higher knowledge of prevention of COVID-19. Finally, the obtained scores were calculated based on 100 and divided into three levels: Low (0–60), moderate (61–80), and high (81–100).

The third part of the instrument were 43 questions about the preventive behaviors against COVID-19 retrieved from the protocols of Disease Control and Prevention (CDC). The scaling of the questionnaire has been organized based on a 4-point Likert scale (never = 0, sometimes = 1, often = 2, and always = 3). The total score of this questionnaire was 129, and the higher score indicated a high level of adherence to preventive behaviors against COVID-19. The obtained scores were calculated based on 100 and divided into three levels: Low (0–60), moderate (61–80), and high (81–100).

In order to assess the content validity, the questionnaire was given to ten professors in nursing and health in Guilan University of Medical Sciences and the requested changes were made. The mean content validity index (CVI) for the questionnaire of knowledge of prevention of COVID-19 was 0.92, and for the preventive behaviors against COVID-19 questionnaire was 0.97. To determine the content validity ratio (CVR), as values ​​higher than 0.62 were approved, therefore, the total questions were accepted.

In order to evaluation of the reliability of the questionnaires, a pilot study was conducted on 20 people. The questionnaires were completed by the participants on two occasions, with an interval of two weeks. Cronbach’s alpha coefficient and intraclass correlation coefficient (ICC) were used respectively to check the internal consistency and test-retest reliability of the questionnaires. The values of Cronbach’s alpha coefficient and ICC for the preventive behaviors against COVID-19 questionnaire were reported as 0.934 and 0.998, respectively. For questionnaire of knowledge of prevention of COVID-19, the Cronbach’s alpha coefficient 0.89 and ICC = 0.95 were obtained, which were at the acceptable levels.

### Data analysis

Data analysis was performed using SPSS for windows, version 16.0 (SPSS Inc., Chicago, IL, USA). Continuous variables were presented as mean (standard deviation (SD)) and median (interquartile range (IQR)) and categorical variables as number (percentage). The Kolmogorov-Smirnov and Shapiro-Wilk tests were used to assess the normality of the data. Spearman’s correlation coefficient was performed to examine the relationship between knowledge and behaviors of prevention of COVID-19. To examine factors associated with high knowledge and good behaviors of prevention of COVID-19, multiple linear regression analysis was performed. In this study the variables of knowledge and behaviors were considered as binary variables, then logistic regression model was used to analyze the data due to its binary nature. Odds ratio (OR) and 95% confidence interval (CI) were calculated. For all analyses, the level of significance was set at 0.05.

## Results

The present study was conducted on 344 rural people. The participants’ personal and social characteristics are presented in Table 1. The mean and standard deviation of participants’ age was 38.64 ± 13.26 years. Most of the participants were female (59.6%), married (78.2%), university educated (35.8%), and housewives (32%). The majority had a history of COVID-19 in themselves or their families (53.5%). Also, only 18.6% had an underlying disease, and 5.2% stated the experience of death due to COVID-19 in their family members (Table 1).

**Table 1:** Personal and social characteristics of the study participants (n = 344)

Variable		N(%)/Mean ± SD
Age (Year)		38.64 ± 13.26
Gender	Male	139 (40.4)
	Female	205 (59.6)
Marital status	Single	66 (19.2)
	Married	169 (78.2)
	Divorced	3 (0.9)
	Widow	6 (1.7)
Number of family members	1	7 (2.0)
	2	60 (17.4)
	3	109 (31.7)
	4	136 (39.5)
	5 and above	32 (9.3)
Education	Under diploma	118 (34.3)
	Diploma	103 (29.9)
	University education	123 (35.8)
Employment status	Self-employed	75 (21.8)
	Farmer	16 (4.7)
	Labor	18 (5.2)
	Employee	63 (18.3)
	Retired	20 (5.8)
	Student-University student	32 (9.3)
	Housewife	110 (32)
	Unemployed	10 (2.9)
Monthly income	Enough to meet life needs	222 (64.5)
	Not enough to meet life needs	122 (35.5)
Underlying disease	Yes	64 (18.6)
	No	280 (81.4)
Getting yourself or your family infected with COVID-19	Yes	184 (53.5)
	No	160 (46.5)
Death of family members due to COVID-19	Yes	18 (5.2)
	No	326 (94.8)
Access to healthcare services	Yes	341 (99.1)
	No	3 (0.9)
Internet access	Yes	307 (89.2)
	No	37 (10.8)
Training by the health center	Yes	256 (74.4)
	No	88 (25.6)
Health workers’ visiting at home	Yes	188 (54.7)
	No	156 (45.3)

Majority of the participants had a moderate level of knowledge about prevention of COVID-19 (47.4%). The mean score and the standard deviation of the knowledge was 73.4 ± 14.3. Median (Interquartile Range) was 72.7 (63.6–81.8) (Table 2).

**Table 2:** Description of score of knowledge of prevention of COVID-19 in the study participants

Variable	Mean ± SD	Median (Interquartile Range)	n (%)		
			Low (0–60)	Moderate (61–180)	High (81–100)
knowledge of prevention of COVID-19	73.4 ± 14.3	72.7 (63.6–81.8)	45 (13.1)	136 (39.5)	(47.4 ) 163

SD: Standard Deviation

In description of questions of the knowledge questionnaire, the highest frequency was related to staying away from crowded places to prevent COVID-19 (96.5%), and the lowest frequency was related to using traditional medicines (24.4%).

Majority of the participants had a good level of preventive behaviors of COVID-19 (44.5%). The mean score and the standard deviation of the preventive behaviors in the participants was 74.8 ± 18.6. Median (Interquartile Range) was 77.9 (65.9–86) (Table 3).

**Table 3:** Description of score of preventive behaviors of COVID-19 in the study participants

Variable	Mean ± SD	Median (Interquartile Range)	n (%)		
			Low (0–60)	Moderate (61–180)	High (81–100)
Preventive behaviors of COVID-19	74.8 ± 18.6	77.9 (65.9–86)	55 (16)	136 (39.5)	153 (4.5)

SD: Standard Deviation

Assessing the questions of the preventive behaviors of COVID-19 showed that the highest frequency of the behaviors (77.6%) was related to avoid touching the inner part of the mask and tissue, and the lowest frequency was related to how to dilute sodium hypochlorite solution (29.1%).

**Fig. 1:**
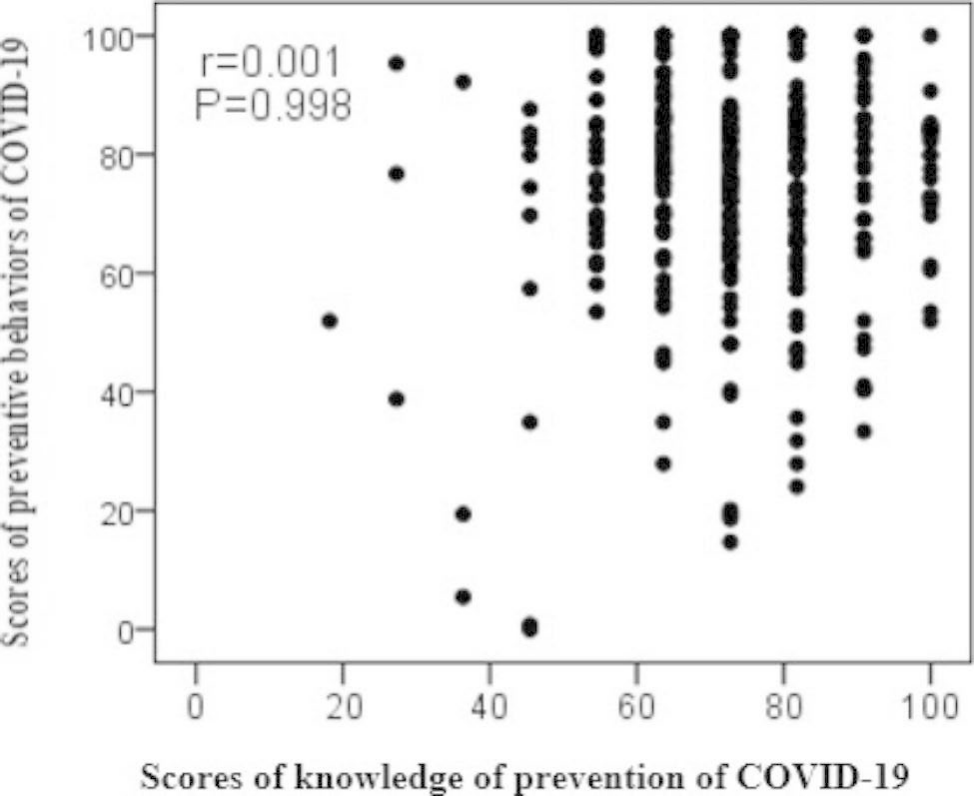
Relationship between scores of knowledge of prevention of COVID-19 with the preventive behaviors of COVID-19

No significant relationship was reported between scores of knowledge of prevention of COVID-19 with the preventive behaviors of COVID-19 (r_s_=0.001, P = 0.998) (Fig. 1).

There were the significant relationship between high level score of knowledge of prevention of COVID-19 with female gender (OR = 2.29, 95% CI: 1.16–4.50, P = 0.016) and the university-educated participants (OR = 6.02, 95% CI: 3.39–10.66, P < 0.001). In contrast, those whose family members had died due to COVID-19, had lower chance for high score of knowledge (P = 0.023, 95% CI = 0.04–0.79, OR = 0.18) (Table 4).

**Table 4:** Factors related to high level of knowledge of prevention of COVID-19 in the study participants using a multiple logistic regression model

Variable		High knowledge	Multiple Regression Analysis	
		**n (%)**	**OR (95% CI)**	***P***
Gender	Male	44 (31.7)	1	
	Female	92 (44.9)	2.29 (1.16–4.50)	0.016
Education	Under diploma	25 (21.2)	1	
	Diploma	35 (34.0)	1.80 (0.88–3.69)	0.109
	University education	76 (61.8)	6.02 (3.39–10.66)	< 0.001
Death of family members due to COVID-19	Yes	2 (11.1)	0.18 (0.04–0.79)	0.023
	No	134 (41.1)	1	

OR: odds ratio; aOR: adjusted odds ratio; CI: confidence interval

There were the significant relationships between high level score of preventive behaviors of COVID-19 with female gender (OR = 1.94, 95% CI: 1.01–3.75, P = 0.049), internet access (OR = 3.22, 95% CI = 1.24–8.33, P = 0.016), training by health workers (OR = 2.02, 95% CI: 1.07–3.81, P = 0.031), visiting by health workers at home (OR = 1.77, 95% CI: 1.03–3.03, P = 0.039), and having experience COVID-19 infection in the participants or their family (OR = 0.49, 95% CI: 0.30–0.81, P = 0.005) (Table 5). Also, no statistically significant relationship was observed between having high level score of knowledge of prevention of COVID-19 and preventive behaviors at a good level (OR = 1.40, 95% CI: 0.82–2.38, P = 0.221). There were not multicollinearity among the study variables.

**Table 5:** Factors related to good level of preventive behaviors of COVID-19 in the study participants using a logistic regression model

Variable		good preventive behaviors	Multiple Regression Analysis	
		**N (%)**	**OR (95% CI)**	***P***
Gender	Male	51 (36.7)	1	
	Female	102 (49.8)	1.94 (1.01–3.75)	0.049
Internet access	Yes	142 (46.3)	3.22 (1.24–8.33)	0.016
	No	11 (29.7)	1	
Training by health workers	Yes	127 (49.6)	2.02 (1.07–3.81)	0.031
	No	26 (29.5)	1	
Visiting by health workers at home	Yes	98 (52.1)	1.77 (1.03–3.03)	0.039
	No	55 (35.3)	1	
Having experience COVID-19 infection in the participants or their family	Yes	69 (37.5)	0.54 (0.35–0.85)	0.005
	No	84 (52.5)	1	

OR: odds ratio; aOR: adjusted odds ratio; CI: confidence interval

## Discussion

The results of the present study showed that the rural people had a moderate level of knowledge and a good level of preventive behaviors of COVID-19, which is consistent with the results of studies of Yue, Mohammadi, and Khazaee-Pool’s [10, 22, 25] and is inconsistent with the findings of the studies of Shahabi et al. and Haque et al. that the participants had not a favorable level of knowledge and performance about COVID-19 [26, 27]. It seems that the reason of the difference can be due to the difference in the time of conducting the studies during the outbreak of COVID-19 and the governmental measures related to the education and awareness of public communities can be effective on their knowledge and the behaviors. Also, the difference in cultural, social, and economic situations can be the another reason. Considering that the study was conducted in villages near the city and distant villages were not included in the study, this can also be related to the level of knowledge of the people participating in this study.

According to the current study, the highest frequency of the knowledge was related to the prevention of COVID-19 using the staying away from crowd places to prevent COVID-19, and the lowest frequency was related to the disease prevention by using traditional medicine. In accordance to the current study, Zhong et al. in their study showed 98.6% of people avoided going to crowded places during the COVID pandemic [3]. The results of the present study is consistent with the results of Chen and Shahabi’s study [1, 26]. These results showed that although level of the knowledge of the rural community was in moderate range, they believed less in traditional medicine and were mostly followed the national guidelines.

The most preventive behaviors of COVID-19 was to avoid touching the inner part of the mask and tissue, and the lowest frequency was related to how to dilute sodium hypochlorite solution. In this regard the, the results of Baghernezhad and Chen’s study are consistent, [1, 28] and the results of Haque’s study [27] are inconsistent with the results of the present research. Also, the results of Shahabi’s study on the ways to prevent disease are the same as the results of the present study [26]. These similar results in studies conducted in Iran can be caused by the common practice of the country’s health system during the COVID-19 pandemic.

In the present study, the participants who were females, those who had more access to the Internet, those who trained by health workers and visited by them at home had the good level of the preventive behaviors. In addition, the chance of having a good level of preventive behavior were 51% lower in people who themselves or their family members were infected with Covid-19 than in those who were not. These findings are consistent with the results of studies of Zhang, Amodan, and Baghernezhad [9, 28, 29] and are inconsistent with the results of Shahabi and Zhong’s study [3, 26]. Also, the results of Khazaee-Pool et al.’s study on the population of Mazandaran province about the association of gender with preventive behaviors against COVID-19, which is consistent with the results of our study. It seems the good level of preventive behaviors in women be because women are more responsible for their family members. It seems that individuals who had access to the Internet, they received more information about the preventive behaviors of COVID-19.

Individuals who were trained by health workers and those who were visited by them at home had the good preventive behaviors. It could be because the rural population has more trust in the educations that provided by health workers, and they are the reliable source for people. Also, in the current study, level of knowledge of the participants about prevention of COVID-19 was higher in women and in the participants that had university education. Chen et al.’s study on urban and rural residents in China showed that individuals with higher education had more knowledge [18]. Moreover, the results of Cvetkovic et al.’s study showed that women and individuals with a higher level of education had higher awareness of COVID-19 [8], which is consistent with our study results. The results of Haque et al.’s study showed that men had the higher knowledge than women [27], which is inconsistent with the results of our study. According to the researcher, knowledge will increase with increasing education, so it seems that this is the reason for the higher knowledge of university-educated individuals. Also, it can be due to the fact that mass medias and scientific websites are the rich resources of information about this issue. In the present study, no significant relationship was observed between level of knowledge about prevention of COVID-19 and preventive behaviors of COVID-19 (P = 0.998, r_s_=0.001), which is consistent with the results of Keyvanlo et al.’s study on women in Iran [5] and is not consistent with the results of Nasirzadeh and Chen’s study [1, 16]. The reason for the inconsistency of the results of the present study with the mentioned studies can be due to the difference in conducting studies during different pics of COVID-19. Li et al. in their study indicated that rural areas had a larger proportion of older cases (> 65 years old) than did the urban areas. It has been reported that rural areas have older populations, on average, and more people with underlying health conditions than urban communities. Additionally, older adults are more likely to be hospitalized. It can justify the good range of preventive behaviors of COVID-19 in the rural communities [30].

One of the limitations of this study is using the self-reporting questionnaire. Also, the present study was conducted in the villages of the suburbs of Rasht city where the distance from the city was short, so the findings may have been influenced by this issue. We suggest that further studies be conducted in rural communities with different ages and cultures and in different geographical environments.

## Conclusion

The results of the current study indicated that the rural people had a moderate level of knowledge and a good level of preventive behaviors of COVID-19. A primary health care approach is essential for education of rural community in epidemic situations in Iran.

### Electronic supplementary material

Below is the link to the electronic supplementary material.

Supplementary Material 1

## Data Availability

The data that support the findings of this study are available from the corresponding author upon reasonable request.

## References

[1] Chen Y, Zhou R, Chen B, Chen H, Li Y, Chen Z (2020). Knowledge, perceived beliefs, and preventive behaviors related to COVID-19 among chinese older Adults: cross-sectional web-based survey. J Med Internet Res.

[2] Ye Y, Wang R, Feng D, Wu R, Li Z, Long C (2020). The recommended and excessive preventive behaviors during the COVID-19 pandemic: a community-based online survey in China. Int J Environ Res and Public Health.

[3] Zhong B-L, Luo W, Li H-M, Zhang Q-Q, Liu X-G, Li W-T (2020). Knowledge, attitudes, and practices towards COVID-19 among chinese residents during the rapid rise period of the COVID-19 outbreak: a quick online cross-sectional survey. Int J Biol Sci.

[4] Imtiaz A, Khan NM, Hossain MA (2022). COVID-19 in Bangladesh: measuring differences in individual precautionary behaviors among young adults. Z Gesundh Wiss.

[5] Keyvanlo ZMFN, Mehri A, Joveini H, Hashemian M (2020). Evaluation of knowledge, attitude and practice of women in Sabzevar about home quarantine to prevent coronavirus. Iran J Obstet Gynecol Infertility.

[6] Rahmanian M, Droudchi A, Zarenejad M, Hatami N, Javadani F, Kalni N (2020). Knowledge, attitude and practice of Jahrom medical students towards the new corona virus (covid 91). Med J Mashhad Univ Med Sci.

[7] Taghrir MH, Borazjani R, Shiraly R (2020). COVID-19 and iranian medical students; a survey on their related-knowledge, preventive behaviors and risk perception. Arch Iran Med.

[8] Cvetković VM, Nikolić N, Radovanović Nenadić U, Öcal A, K Noji E, Zečević M (2020). Preparedness and preventive behaviors for a pandemic disaster caused by COVID-19 in Serbia. Int J Environ Res Public Health.

[9] Amodan BO, Bulage L, Katana E, Ario AR, Fodjo JNS, Colebunders R (2020). Level and determinants of adherence to COVID-19 preventive measures in the First Stage of the outbreak in Uganda. Int J Environ Res Public Health.

[10] Mohammadi S, Nakhaeizadeh A (2021). Assessing the level of Engagement in Preventive Behaviors and COVID-19 related anxiety in iranian adults. Avicenna J Nurs Midwifery Care.

[11] World Health Organization (2022). Coronavirus disease (COVID-19) Weekly Epidemiological Update and Weekly operational update.

[12] Ramadhani PR, Syamsyudin A, editors. Early Childhood Sex Education in Coastal Areas. 1st Paris Van Java International Seminar on Health, Economics, Social Science and Humanities (PVJ-ISHESSH 2020). Atlantis Press; 2021.

[13] Tajerimoghadam Z, Yazdanpanah M (2020). Analysis of preventive behaviors against corona virus case: rural areas of Dashtestan city. Q J Space Econ Rural Dev.

[14] Ma L, Liu H, Tao Z, Jiang N, Wang S, Jiang X, Knowledge (2020). Beliefs/Attitudes, and practices of rural residents in the prevention and control of COVID-19: an online questionnaire survey. Am J Trop Med Hyg.

[15] Liu PL, Cyberpsychology (2020). Behav Social Netw.

[16] Nasirzadeh M, Aligol M (2020). Assessmentof knowledge, attitude, and factors Associated with the preventive behaviors of Covid-19 in Qom, Iran, in 2020. Qom Univ Med Sci J.

[17] Li S, Feng B, Liao W, Pan W (2020). Internet use, risk awareness, and demographic characteristics associated with engagement in preventive behaviors and testing: cross-sectional survey on COVID-19 in the United States. J med Internet Res.

[18] Chen X, Chen H (2020). Differences in preventive behaviors of COVID-19 between urban and rural residents: lessons learned from a cross-sectional study in China. Int J Environ Res Public Health.

[19] Banda J, Dube A, Brumfield S, Amoah A, Crampin A, Reniers G (2020). Knowledge and behaviors related to the COVID-19 pandemic in Malawi. medRxiv.

[20] World Health Organization. Coronavirus disease (COVID-19) [Internet]. [cited https://www.who.int/health-topics/coronavirus#tab=tab_3].

[21] Centers for Disease Control and Prevention. Coronavirus Disease 2019 [Internet]. [cited https://www.cdc.gov/coronavirus/2019-ncov/prevent-getting-sick/prevention.html].

[22] Khazaee-Pool M, Shahrvsand S, Naghibi SA (2020). Predicting Covid-19 preventive behaviors based on Health Belief Model: an internet-based study in Mazandaran Province, Iran. J Mazandaran Univ Med Sci.

[23] Glaser D. Child sexual abuse. 2nd ed. Macmillan International Higher Education; 1993.

[24] Tabachnick BGFL (2013). Using multivariate statistics.

[25] Yue S, Zhang J, Cao M, Chen B (2021). Knowledge, attitudes and practices of COVID-19 among urban and rural residents in China: a cross-sectional study. J Community Health.

[26] Shahabi N, Takhti HK, Azad MH, Rad RE, Ghaffari HR, Mohseni S (2022). Knowledge, attitude, and preventive behaviors of Hormozgan residents toward COVID-19, one month after the epidemic in Iran. Z Gesundh Wiss.

[27] Haque T, Hossain KM, Bhuiyan MMR, Ananna SA, Hussain MA, Islam MR et al. Knowledge, attitude and practices (KAP) towards COVID-19 and assessment of risks of infection by SARS-CoV-2 among the bangladeshi population: an online cross sectional survey. 2020:10.21203/rs.3.rs-4562/v2.

[28] Baghernezhad Hesary F, Salehiniya H, Miri M, Moodi M (2021). Investigating preventive behaviors toward COVID-19 among iranian people. Front Public Health.

[29] Zhang M, Li Q, Du X, Zuo D, Ding Y, Tan X (2020). Health Behavior toward COVID-19: the role of demographic factors, knowledge, and attitude among Chinese College Students during the Quarantine Period. Asia Pac J Public Health.

[30] Li Y, Hu T, Gai X, Zhang Y, Zhou X (2021). Transmission Dynamics, Heterogeneity and Controllability of SARS-CoV-2: a rural–urban comparison. Int J Environ Res Public Health.

