# Supplementary material for: Knowledge and behaviors of prevention of COVID-19 and the related factors in the rural population referred to the health centers: a cross-sectional study
Source: BMC Nurs. 2023 Dec 13;22:474. doi: 10.1186/s12912-023-01469-5 (PMC10717995; doi:10.1186/s12912-023-01469-5)
Supplement: Supplementary file 1 — Supplementary Material 1 [file 12912_2023_1469_MOESM1_ESM.docx]

**A: Socio- demographic variables**

- Age: …..(year)
- Gender : male 🗆 female 🗆
- Marital status: single 🗆 married 🗆 divorced 🗆 widowed 🗆
- Number of family members: …..
- Educational level: illiterate 🗆 under diploma 🗆 diploma 🗆 academic education 🗆
- Occupation: self- employed 🗆 farmer 🗆 worker 🗆 government's employee🗆 Retired 🗆 student 🗆 housewife 🗆 unemployed 🗆
- family income status (monthly): adequate 🗆 inadequate 🗆
- Having a underlying disease: Yes 🗆 No 🗆

If your answer is positive please state type of your disease…….

- History of contracting COVID-19 in you or other family members: Yes 🗆 No 🗆
- Death of family members due to COVID-19: Yes 🗆 No 🗆
- Have you received the COVID-19 vaccine? Yes 🗆 No 🗆
- Do you have access to healthcare and medical services in your village? Yes 🗆 No 🗆
- Do you have the possibility to access and use the Internet? Yes 🗆 No 🗆
- Have you been trained by the health center about corona disease and ways to prevent it? Yes 🗆 No 🗆
- From which of the following sources have you received information about COVID-19? TV 🗆 website 🗆 SMS 🗆 magazine 🗆 friends 🗆 others 🗆
- Have the paramedics or other healthcare workers visited your home to check the status of the corona virus? Yes 🗆 No 🗆

**B: Knowledge about COVID-19** (Yes=1 No=0)

1. Is the COVID-19 transmitted from one person to another? Yes 🗆 No 🗆
2. What is the main way of transmission of COVID-19? Respiratory droplets 🗆 water 🗆 foods 🗆 feces 🗆
3. Can diarrhea and abdominal pain be symptoms of COVID-19? Yes 🗆 No 🗆
4. How many days after contact with the virus can the symptoms of COVID-19 be transmitted?
   1. 2-5 days 🗆 2-14 days 🗆 < 2 days 🗆 I don’t know 🗆
5. Is using a simple surgical mask effective in preventing COVID-19? Yes 🗆 No 🗆
6. Is staying away from gatherings effective in preventing COVID-19? Yes 🗆 No 🗆
7. Can all age groups be affected by COVID-19? Yes 🗆 No 🗆
8. Does a person who has been infected with COVID-19 once, will not be infected with this disease again? Yes 🗆 No 🗆
9. Can regularly washing the nose with salt prevent infection with COVID-19?

Yes 🗆 No 🗆

1. Can regularly washing the nose with salt prevent infection with COVID-19?

Yes 🗆 No 🗆

1. Is there a definitive treatment for COVID-19?

Yes 🗆 No 🗆

**C: Preventive behaviors of COVID-19 questionnaire**

| NO. | ***Items*** | Always | Often | Sometimes | Never |
| --- | --- | --- | --- | --- | --- |
| 1 | When I cough or sneeze, I put a tissue or my elbow in front of my mouth and nose. | 3 | 2 | 1 | 0 |
| 2 | I refrain from touching the inside of the tissue that I sneezed and coughed into. | 3 | 2 | 1 | 0 |
| 3 | After sneezing or coughing, I wash my hands. | 3 | 2 | 1 | 0 |
| 4 | I put the mask on my face so that it completely covers my mouth, nose and chin.. | 3 | 2 | 1 | 0 |
| 5 | If the mask gets wet, I change it. | 3 | 2 | 1 | 0 |
| 6 | Whenever I touch the mask, I wash or disinfect my hands. | 3 | 2 | 1 | 0 |
| 7 | To remove the mask from my face without touching it, I grab the band or elastic and throw it away. | 3 | 2 | 1 | 0 |
| 8 | After each use of the disposable mask, I throw it away. | 3 | 2 | 1 | 0 |
| 9 | If we use a fabric mask, we wash it after each use. | 3 | 2 | 1 | 0 |
| 10 | After removing the mask every time, I wash my hands with soap and water or disinfect them with alcohol. | 3 | 2 | 1 | 0 |
| 11 | I avoid touching my eyes, nose and mouth. | 3 | 2 | 1 | 0 |
| 12 | I don't shake hands with others. | 3 | 2 | 1 | 0 |
| 13 | I don't kiss others. | 3 | 2 | 1 | 0 |
| 14 | I wash my hands regularly with soap and water. | 3 | 2 | 1 | 0 |
| 15 | I wash my hands for at least 20 seconds. | 3 | 2 | 1 | 0 |
| NO. | ***Items*** | Always | Often | Sometimes | Never |
| 16 | After entering the house, before touching anything, we wash our hands with soap and water. | 3 | 2 | 1 | 0 |
| 17 | To disinfect the surfaces and door handles of the house, I clean it with detergents. | 3 | 2 | 1 | 0 |
| 18 | After entering the house, I hang my outer clothes away from other things. | 3 | 2 | 1 | 0 |
| 19 | After entering the house, I disinfect all my personal belongings (including mobile phone, key chain, car switch, wallet, etc.). | 3 | 2 | 1 | 0 |
| 20 | I disinfect the items I bought from outside before using them or wash them with detergents. | 3 | 2 | 1 | 0 |
| 21 | When disinfecting my hands, I rub the palms, between the fingers and the tips until the alcohol dries. | 3 | 2 | 1 | 0 |
| 22 | To ensure the effectiveness of 70% alcohol solution or hand sanitizer solution, I buy it from the pharmacy. | 3 | 2 | 1 | 0 |
| 23 | I use diluted sodium hypochlorite to disinfect surfaces. | 3 | 2 | 1 | 0 |
| 24 | I dilute sodium hypochlorite in a plastic container. | 3 | 2 | 1 | 0 |
| 25 | To dilute sodium hypochlorite to disinfect surfaces and objects, I pour one cup of it in 5 cups of cold water. | 3 | 2 | 1 | 0 |
| 26 | After pouring the disinfectant solution on the surfaces, I let the solution remain on the surfaces for 10 minutes. | 3 | 2 | 1 | 0 |
| 27 | I use a mask when leaving the house and in all public places. | 3 | 2 | 1 | 0 |
| 28 | When leaving home, I take 70% alcohol solution with me. | 3 | 2 | 1 | 0 |
| 29 | I avoid going to crowded places. | 3 | 2 | 1 | 0 |
| 30 | I leave the house only when necessary. | 3 | 2 | 1 | 0 |
| 31 | I keep a distance of 1.5 to 2 meters with others. | 3 | 2 | 1 | 0 |
| 32 | I refuse to attend family gatherings, parties and any kind of ceremony. | 3 | 2 | 1 | 0 |
| 33 | I open the doors and windows when I am required to be in a closed space. | 3 | 2 | 1 | 0 |
| 34 | I do not use public transport as much as possible. | 3 | 2 | 1 | 0 |
| 35 | If I need public transport, I use a mask. | 3 | 2 | 1 | 0 |
| 36 | I refuse to ride crowded public transport.... | 3 | 2 | 1 | 0 |
| 37 | I open the window when I get on public transport | 3 | 2 | 1 | 0 |
| NO. | ***Items*** | Always | Often | Sometimes | Never |
| 38 | I use bank cards not bills when shopping. | 3 | 2 | 1 | 0 |
| 39 | When shopping, I swipe the card myself in the card reader to pay. | 3 | 2 | 1 | 0 |
| 40 | I use gloves or tissue when working with ATM banking. | 3 | 2 | 1 | 0 |
| 41 | After using bank card or money, I disinfect my hand or card. | 3 | 2 | 1 | 0 |
| 42 | If I use the elevator, I use gloves or tissue to press its buttons. | 3 | 2 | 1 | 0 |
| 43 | I avoid entering the crowded elevator | 3 | 2 | 1 | 0 |
